# Clinical iron deficiency disturbs normal human responses to hypoxia

**DOI:** 10.1172/JCI85715

**Published:** 2016-05-03

**Authors:** Matthew C. Frise, Hung-Yuan Cheng, Annabel H. Nickol, M. Kate Curtis, Karen A. Pollard, David J. Roberts, Peter J. Ratcliffe, Keith L. Dorrington, Peter A. Robbins

**Affiliations:** 1 University of Oxford, Department of Physiology, Anatomy and Genetics, Oxford, United Kingdom.; 2 Oxford University Hospitals NHS Foundation Trust, Oxford Centre for Respiratory Medicine, Churchill Hospital, Oxford, United Kingdom.; 3 University of Oxford, Nuffield Department of Clinical Laboratory Sciences, and National Health Service Blood and Transplant Oxford Centre, John Radcliffe Hospital, Oxford, United Kingdom.; 4 University of Oxford, Nuffield Department of Medicine, Henry Wellcome Building for Molecular Physiology, Old Road Campus, Headington, Oxford, United Kingdom.

## Abstract

**BACKGROUND.** Iron bioavailability has been identified as a factor that influences cellular hypoxia sensing, putatively via an action on the hypoxia-inducible factor (HIF) pathway. We therefore hypothesized that clinical iron deficiency would disturb integrated human responses to hypoxia.

**METHODS.** We performed a prospective, controlled, observational study of the effects of iron status on hypoxic pulmonary hypertension. Individuals with absolute iron deficiency (ID) and an iron-replete (IR) control group were exposed to two 6-hour periods of isocapnic hypoxia. The second hypoxic exposure was preceded by i.v. infusion of iron. Pulmonary artery systolic pressure (PASP) was serially assessed with Doppler echocardiography.

**RESULTS.** Thirteen ID individuals completed the study and were age- and sex-matched with controls. PASP did not differ by group or study day before each hypoxic exposure. During the first 6-hour hypoxic exposure, the rise in PASP was 6.2 mmHg greater in the ID group (absolute rises 16.1 and 10.7 mmHg, respectively; 95% CI for difference, 2.7–9.7 mmHg, *P* = 0.001). Intravenous iron attenuated the PASP rise in both groups; however, the effect was greater in ID participants than in controls (absolute reductions 11.1 and 6.8 mmHg, respectively; 95% CI for difference in change, –8.3 to –0.3 mmHg, *P* = 0.035). Serum erythropoietin responses to hypoxia also differed between groups.

**CONCLUSION.** Clinical iron deficiency disturbs normal responses to hypoxia, as evidenced by exaggerated hypoxic pulmonary hypertension that is reversed by subsequent iron administration. Disturbed hypoxia sensing and signaling provides a mechanism through which iron deficiency may be detrimental to human health.

**TRIAL REGISTRATION.** ClinicalTrials.gov (NCT01847352).

**FUNDING.** M.C. Frise is the recipient of a British Heart Foundation Clinical Research Training Fellowship (FS/14/48/30828). K.L. Dorrington is supported by the Dunhill Medical Trust (R178/1110). D.J. Roberts was supported by R&D funding from National Health Service (NHS) Blood and Transplant and a National Institute for Health Research (NIHR) Programme grant (RP-PG-0310-1004). This research was funded by the NIHR Oxford Biomedical Research Centre Programme.

## Introduction

Cellular and integrated physiological responses to variations in oxygen availability are regulated in metazoan organisms by transcription factors known as hypoxia-inducible factors (HIFs) (1, 2). HIF is active as a transcription factor when in a heterodimeric form consisting of 1 HIF-α and 1 HIF-β subunit (3). These heterodimers bind to hypoxia-response elements (HREs) in the genome and control the expression of many hundreds of genes, including those central to the regulation of erythropoiesis (4), angiogenesis (5), and metabolism (6). The consequences of targeted genetic disruption of the HIF pathway in animal models demonstrate the importance of HIF in regulating these processes, and also indicate that HIF is a key regulator of pulmonary vascular and respiratory physiology (7–13). Spontaneously occurring mutations in humans confirm this to be the case, with genetic upregulation of the pathway resulting in polycythemia, pulmonary arterial hypertension, abnormal ventilatory drive, and impaired skeletal muscle oxidative phosphorylation (14–18). Additionally, in some human populations resident for thousands of years at high altitude, there is evidence for natural selection of HIF pathway gene variants associated with downregulation of hypoxia sensing (19–21).

The basis for the oxygen-sensitivity of the pathway is that the HIF-α subunit can undergo hydroxylation at 3 amino acid residues by a group of enzymes called HIF hydroxylases (22). HIF-α may be hydroxylated at 2 specific proline residues by prolyl-hydroxylase domain enzymes (PHDs). Hydroxylation at either site marks HIF-α for polyubiquitination and proteasomal degradation (23–25). Hydroxylation at a single asparagine residue, by an enzyme known as factor inhibiting HIF (FIH), does not promote HIF-α degradation but instead blocks recruitment of coactivators of transcription to the HIF-HRE complex (26–28). These hydroxylation reactions absolutely require dioxygen, such that as oxygen tension falls, the rate of HIF-α hydroxylation is slowed. HIF-α thus accumulates, leading to greater abundance of HIF heterodimers, which are able to recruit transcriptional coactivators and control HRE-regulated genes. The HIF-β subunit, in contrast, is constitutively expressed and is not oxygen-regulated.

The PHDs and FIH are members of a superfamily of 2-oxoglutarate–dependent dioxygenases, which includes members with diverse biological roles, from collagen synthesis to histone demethylation (29). These enzymes all share the requirement for a single ion of ferrous iron at their active sites, which is involved in electron transfer (30), giving rise to the possibility that HIF hydroxylase activity might be sensitive to intracellular iron availability (31, 32). Indeed, prior to the characterization of the HIF hydroxylases, both sensitivity to iron chelation with desferrioxamine (DFO) and sensitivity to Fe^2+^ substitution with Ni^2+^ or Co^2+^ ions were used as indicators of whether a pathway may be regulated by HIF (31, 33). Cell culture experiments have subsequently confirmed an effect of iron availability on HIF via altered HIF hydroxylase function (34–37).

An unusual aspect of human iron homeostasis is that there is no regulated mechanism for the excretion of excess iron (38). An apparently paradoxical consequence is that iron deficiency is extremely common (39), since iron uptake must be tightly regulated to prevent excess iron accumulation. Additionally, a state of iron sequestration may exist as a result of inflammation in the setting of chronic disease, even in individuals with adequate total body iron stores (40, 41). It is at present unknown, however, whether these clinical variations in iron status have an effect on HIF hydroxylase activity that translates into significant consequences for the pathobiology of human oxygen sensing. This question is of very considerable importance for human health and disease because conditions in which hypoxia plays a key role are very common global causes of morbidity and mortality (2, 42), and iron deficiency affects more individuals worldwide than any other medical condition (43).

In the present study, we set out to determine whether there exists a direct effect of clinical iron deficiency in modulating responses to hypoxia in humans. We focused on the pulmonary circulation as a model system for investigating the interaction between iron and oxygen sensing and signaling, because acute manipulation of iron bioavailability has been shown markedly to affect the hypoxic behavior of the pulmonary vasculature (44, 45). In contrast to the systemic circulation, the response of the pulmonary vasculature to hypoxia is to vasoconstrict (46), and the magnitude of this response during global alveolar hypoxia can be determined from the consequent rise in pulmonary artery systolic pressure (PASP) (47, 48).

## Results

### Baseline characteristics of iron-deficient and iron-replete groups.

**Figure 1:**
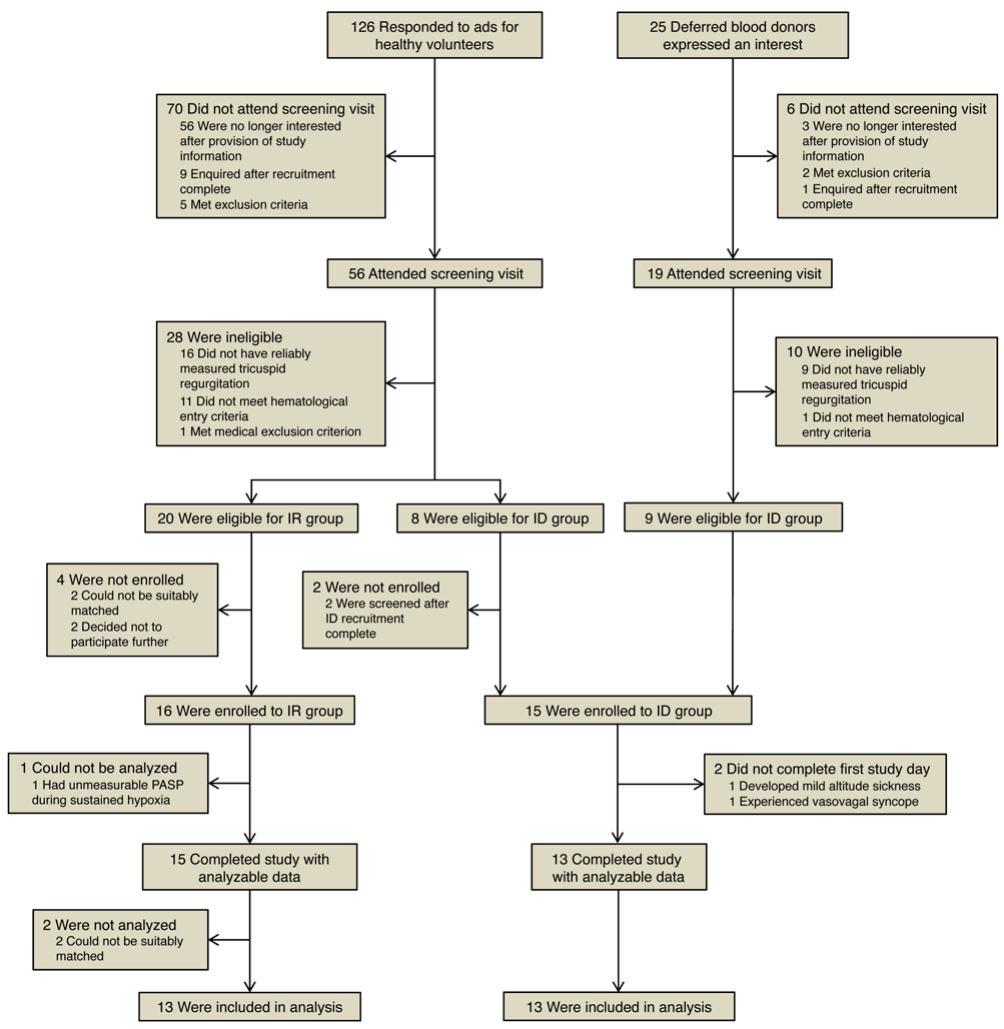
Study recruitment flow diagram. During the period of recruitment there were 25 expressions of interest from deferred blood donors and 126 responses to advertisements for healthy volunteers. In total, 16 participants were enrolled to the IR group and 15 to the ID group. Two female participants in the ID group were withdrawn during the first hypoxic exposure. The first developed headache and nausea consistent with altitude sickness. The second experienced vasovagal syncope. Both recovered promptly without sequelae on return to air. One participant in the IR group had echocardiographic data during hypoxia that precluded accurate measurement of PASP, despite a successful screening visit. No suitable ID participants presented themselves as matches for 2 young males recruited to the IR group early in the course of the study; data for these individuals were not analyzed. Thus, 13 ID individuals completed the study and were matched with an equal number of IR controls in the per-protocol analysis; there were no missing data.

Thirteen age- and sex-matched pairs of iron-deficient (ID) and iron-replete (IR) healthy individuals were identified as illustrated in Figure 1. Characteristics of these participants are given in Table 1. There were no significant differences in BMI or spirometric parameters between groups. Mean ferritin in the ID group was 6.4 μg/l and transferrin saturation 8.4%, indicating profound absolute iron deficiency. Corresponding values for the IR group were 66.9 μg/l and 29.2%, respectively, consistent with physiologically normal iron stores. Plasma soluble transferrin receptor (sTfR) was significantly higher in the ID group; the mean exceeded the upper limit of normal of 28.1 nmol/l for the healthy population (49), implying a significant unmet tissue iron demand. None of the IR group had an elevated sTfR. Hepcidin, a peptide hormone central to iron homeostasis, was very heavily suppressed in the ID group compared with their IR counterparts.

### Hypoxic pulmonary hypertension.

**Figure 2:**
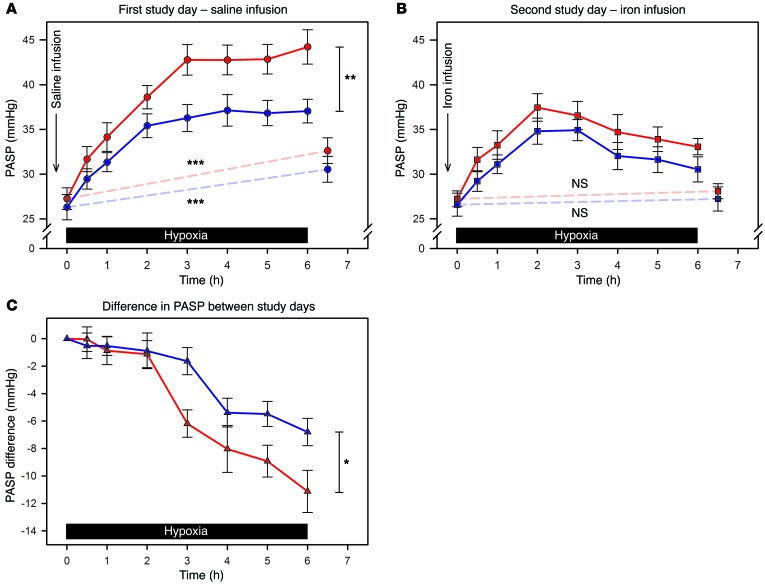
PASP responses to hypoxia. (**A**) First study day (saline infusion). (**B**) Second study day (iron infusion). (**C**) Difference in response between study days. Responses for the ID group are shown in red, and those for the IR group are shown in blue (data are means ± SEM; *n* = 13 in each group). Solid black bars indicate the 6-hour periods of eucapnic hypoxia. Continuous lines represent responses during hypoxia. Broken lines indicate the change in air-breathing PASP induced by the 6-hour period of hypoxia, which reflects the degree of acclimatization of the pulmonary vasculature. On the first study day, the increase in PASP during hypoxia was significantly greater in the ID group. On the second study day, following iron infusion, the increase in PASP was significantly attenuated in both groups. Euoxic PASP was significantly elevated following exposure to 6 hours of hypoxia in both groups on the first study day, but this effect was abolished by prior administration of i.v. iron on the second day. Panel **C** illustrates that the effect on PASP of prior iron administration was minimal for the first 2 hours of hypoxia. Thereafter, iron administration attenuated hypoxic pulmonary hypertension to a greater extent in the ID group. Asterisks indicate significance of comparisons between or within groups: *P < 0.05; **P < 0.01; ***P < 0.001; NS, not significant (mixed-effects model).

Figure 2 illustrates hypoxic PASP responses for both groups. PASP prior to each hypoxic exposure did not differ across group or study day (ID vs. IR: mean 27.3 vs. 26.3 mmHg, on first day; 27.2 vs. 26.6 mmHg, on second day; *P* = 0.59 for group; *P* = 0.79 for study day; *P* = 0.75 for interaction). After 6 hours of hypoxia on the first day, the ID group reached a mean PASP of 44.2 mmHg compared with 37.0 mmHg in the IR group. Hypoxia-induced pulmonary hypertension was therefore 6.2 mmHg greater in the ID group (95% CI, 2.7–9.7 mmHg, *P* = 0.001) (Figure 2A). On the second day, following i.v. iron, the PASP increase diminished in both groups (Figure 2B). At 6 hours, the mean in the ID group was 33.1 mmHg and in the IR group 30.5 mmHg, representing absolute reductions in the rise during hypoxia of 11.1 and 6.8 mmHg, respectively (Figure 2C). The magnitude of the absolute reduction seen in the ID group was significantly greater than that in the IR group (–4.3 mmHg, 95% CI, –8.3 to –0.3 mmHg, *P* = 0.035). After 30 minutes of euoxia following cessation of hypoxia on the first day, euoxic PASP was elevated in both groups compared with values prior to hypoxia (mean increase in euoxic PASP: ID, 5.4 mmHg; IR, 4.2 mmHg; *P* < 0.001 for both groups), reflecting acclimatization of the pulmonary vasculature (Figure 2A). On the second day, with i.v. iron, an increase in euoxic PASP 30 minutes after hypoxia was no longer evident (Figure 2B). The difference between study days was significant (reduction in euoxic PASP at 30 minutes after hypoxia compared with first day: ID, –4.5 mmHg; IR, –3.6 mmHg; *P* < 0.001 for both groups).

### Hypoxic ventilatory responses and peripheral oxyhemoglobin saturation.

**Figure 3:**
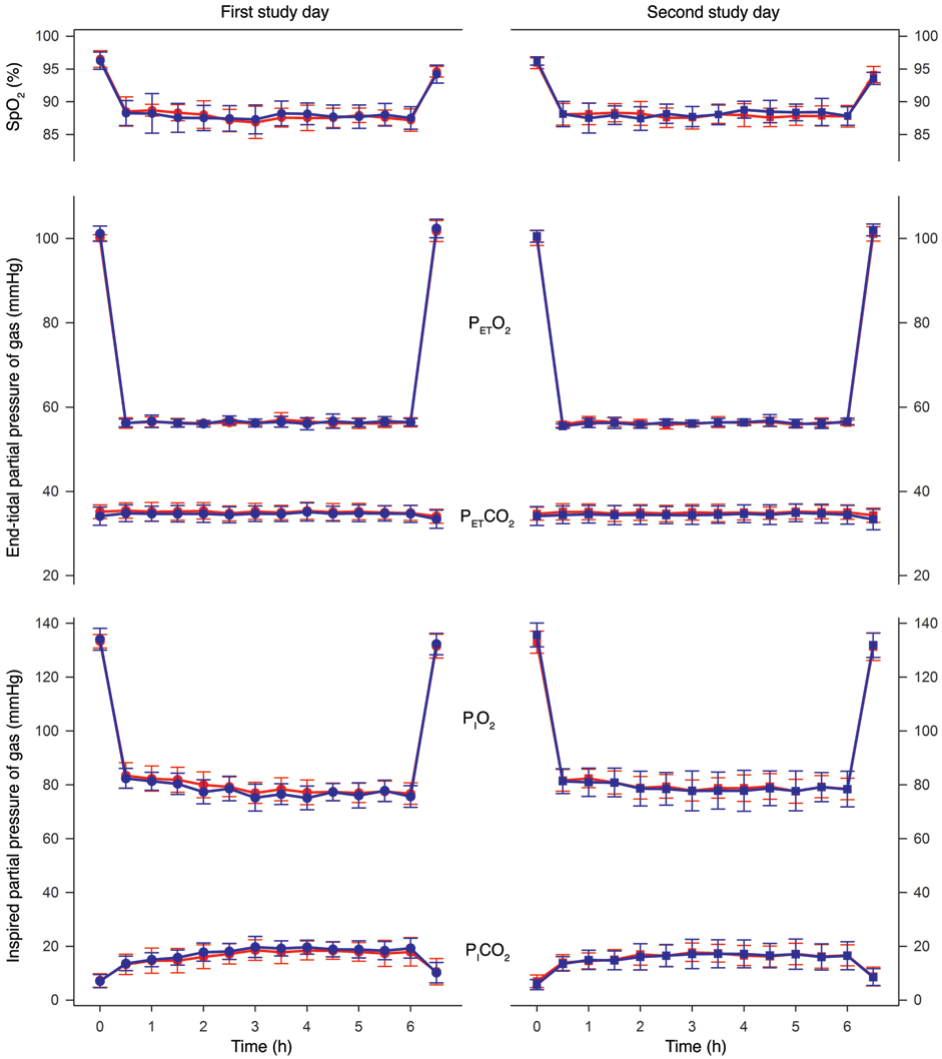
Peripheral oxyhemoglobin saturation and partial pressures of oxygen and carbon dioxide on each study day. Left, first study day (circles); right, second study day (squares). Top panel, peripheral oxyhemoglobin saturation (SpO_2_); middle panel, end-tidal partial pressure of oxygen (P_ET_O_2_) and carbon dioxide (P_ET_CO_2_); bottom panel, inspired partial pressure of oxygen (P_I_O_2_) and carbon dioxide (P_I_CO_2_). Data for the ID group are shown in red and for the IR group in blue (*n* = 13 in each group); data are means ± SD based on time-averaged continuous recordings.

The starting end-tidal partial pressure of carbon dioxide (P_ET_CO_2_) did not differ between the ID and IR groups on either study day, nor on the different days within groups (35.9 vs. 35.4 mmHg, respectively, on the first day; 35.5 vs. 34.9 mmHg, respectively, on the second day; *P* = 0.45 for group; *P* = 0.052 for study day; *P* = 0.77 for interaction). Figure 3 shows the end-tidal and inspired partial pressures of gases, as well as the peripheral oxyhemoglobin saturation (SpO_2_), in both groups for each study day. The mean end-tidal partial pressure of oxygen (P_ET_O_2_) during the hypoxic exposures did not vary between groups or study days (*P* = 0.96 for group; *P* = 0.22 for study day; *P* = 0.74 for interaction), confirming that uniform hypoxic stimuli were delivered. Similarly, the mean SpO_2_ during the hypoxic exposures did not vary between groups or study days (*P* = 0.78 for group; *P* = 0.33 for study day; *P* = 0.83 for interaction). The inspired partial pressure of carbon dioxide (P_I_CO_2_) — an index of ventilation when P_ET_CO_2_ is clamped to maintain eucapnic conditions — did not differ between groups or study days at 30 minutes (approximating to the acute hypoxic ventilatory response) (*P* = 0.94 for group; *P* = 0.61 for study day; *P* = 0.50 for interaction) or at 6 hours (*P* = 0.67 for group; *P* = 0.08 for study day; *P* = 0.51 for interaction).

### Cardiac output responses to hypoxia.

Starting euoxic cardiac output (CO) did not differ between the ID and IR groups on either study day, nor on the different days within groups (mean 5.6 vs. 5.3 l/min, respectively, on first study day; 5.6 vs. 5.1 l/min, respectively, on second study day; *P* = 0.45 for group; *P* = 0.48 for study day; *P* = 0.57 for interaction). CO increased during hypoxia in both groups on both days (first study day 1.1 and 0.8 l/min, respectively; second study day 0.8 and 1.0 l/min, respectively; *P* < 0.001 for each group, each day). In contrast to PASP, the CO response to hypoxia did not differ between groups or study days, and there was no differential effect of i.v. iron between groups (*P* = 0.29 for group; *P* = 0.91 for study day; *P* = 0.25 for interaction).

### Iron and oxygen-sensing and signaling pathways.

Table 2 gives measurements of serum erythropoietin, plasma hepcidin, and serum IL-6 made in each group at the start of both study days (immediately prior to each infusion), and at the conclusion of each 6-hour hypoxic exposure. In all 3 cases, there were no significant within-group differences in mean starting values between the first and second study days.

Serum erythropoietin was higher before the hypoxic exposure in the ID than in the IR group at the start of both study days (ID vs. IR: mean 25.4 vs. 7.5 mIU/ml, on first day; 20.1 vs. 6.8 mIU/ml, on second day; *P* = 0.018 for group; *P* = 0.08 for study day; *P* = 0.16 for interaction). In the ID group, euoxic serum erythropoietin on the first study day correlated strongly with both serum ferritin and hemoglobin concentration (Spearman’s ρ = –0.69, *P* = 0.009 for both relationships); these latter 2 variables also correlated with one another (ρ = 0.65, *P* = 0.02). In contrast, euoxic serum erythropoietin in the IR group on the first study day showed no correlation with either serum ferritin (ρ = 0.02, *P* = 0.92) or hemoglobin concentration (ρ = –0.165, *P* = 0.58), though these latter 2 variables did correlate with one another (ρ = 0.64, *P* = 0.02), as in the ID group. Exposure to hypoxia significantly increased serum erythropoietin on the first study day irrespective of iron status; however, the effect was greater in the ID group. Findings on the second day were similar.

Plasma hepcidin was lower before the hypoxic exposure in the ID than in the IR group at the start of both study days (ID vs. IR: mean 4.5 vs. 20.1 μg/l, on first day; 3.2 vs. 18.9 μg/l, on second day; *P* = 0.005 for group; *P* = 0.63 for study day; *P* = 0.97 for interaction). Exposure to hypoxia significantly increased plasma hepcidin on the first study day; however, the effect was attenuated in the ID group. On the second day, the magnitude of the rise in plasma hepcidin during hypoxia was significantly increased following i.v. iron, but the size of this effect was similarly attenuated in the ID group.

Serum IL-6 prior to each hypoxic exposure did not differ by group or study day (ID vs. IR: mean 0.6 vs. 1.1 ng/l, on first day; 0.7 vs. 0.9 ng/l, on second day; *P* = 0.21 for group; *P* = 0.81 for study day; *P* = 0.53 for interaction). Serum IL-6 significantly increased during exposure to hypoxia on the first study day, but there was no difference in the rise between groups. Findings on the second day were similar.

## Discussion

In recent years, considerable evidence has emerged of the harm associated with iron deficiency in cardiopulmonary diseases and of a benefit from administering i.v. iron in these conditions. Iron deficiency appears to be particularly important in pulmonary vascular disease (50–53) but has also been linked to poorer outcomes in chronic heart failure (54–56), acute heart failure (57), and chronic obstructive pulmonary disease (58). Historically, anemia has been considered to be the most significant consequence of iron deficiency (38). Although iron-deficient, the patients in these recent studies were not necessarily anemic; iron deficiency was an independent risk factor for poor outcome. Equally, though hemoglobin did not invariably rise with the provision of iron, it was clear that treatment had significant clinical benefits (55, 56, 59). These studies are remarkable for the absence of any clear mechanistic explanation for the profound effects of iron deficiency and supplementation.

The hypothesis of the present study was that iron deficiency would act significantly to alter human responses to hypoxia. This hypothesis was founded not simply on an understanding of the molecular biology of the HIF pathway, but on the observed effects of iron chelation in cell culture and intact humans. First, iron chelation with DFO was shown to induce HIF activity and erythropoietin mRNA expression in vitro with a time course very similar to that of hypoxia (60). Subsequently, infusion of DFO was found to elevate PASP (47) and circulating erythropoietin levels (61) in healthy humans breathing air, and also to augment the PASP rise seen in response to a brief hypoxic challenge (45). Conversely, acute i.v. iron loading attenuated both the rise in PASP during prolonged hypoxia (62) and the augmented hypoxic pulmonary vasoconstriction usually seen afterward (45). Lacking, however, has been any demonstration of the effects of clinical iron deficiency on hypoxia-sensing and signaling mechanisms.

A major difficulty in extrapolating the findings of work using iron chelation is that acute infusion of DFO is very different indeed from the insidious development of iron deficiency seen in clinical practice. DFO permeates cell membranes slowly, preferentially depletes hepatic and reticuloendothelial iron, and cannot effectively chelate iron bound to circulating transferrin (63); thus the pattern of tissue iron depletion from an acute infusion of DFO is likely to differ considerably from that seen in naturally occurring iron deficiency. Moreover, DFO has actions aside from iron chelation; it participates in oxidation and reduction reactions, and has free-radical scavenging properties (64). ROS are implicated in many hypoxia-sensing and signaling pathways (65), so DFO may interfere directly with human responses to hypoxia independent of any effect on iron. The present study overcomes these problems and moves from short-term experimental manipulation of iron bioavailability, to demonstrating that the predicted effects of iron deficiency on responses to hypoxia are significant for human health.

We found striking exaggeration of hypoxic pulmonary hypertension in healthy individuals with iron deficiency. After only 6 hours of moderate alveolar hypoxia, the ID group showed a mean rise in PASP that was in excess of 50% greater than that seen in IR controls. The hemoglobin concentration ([Hb]) in the ID group was, as expected, somewhat lower than that in the IR group, and it must be considered whether this difference could have contributed to the findings. Experimental evidence from a range of settings indicates that a lower [Hb] acts to attenuate hypoxic pulmonary vasoconstriction, rather than to augment it. First, detailed animal experiments using perfused rabbit lungs (66) and intact dogs (67) indicate that a lower [Hb] impairs hypoxic pulmonary vasoconstriction. Second, isovolemic hemodilution of high-altitude residents with pulmonary hypertension secondary to chronic hypobaric hypoxia quickly brings about a fall in PASP and pulmonary vascular resistance (68, 69). Thirdly, in patients with severe chronic hypoxemic lung disease, gradual reduction of [Hb] by repeated small-volume venesection results in a significant fall in mean pulmonary artery pressure and pulmonary vascular resistance (70). Taking these observations together, it is very difficult indeed to see how a lower [Hb] could account for the much greater hypoxic PASP rise seen in the ID group, since any influence appears to act in entirely the opposite direction. Indeed, some investigators argue that in examining the behavior of the pulmonary circulation during hypoxia, a correction should be applied for this attenuating effect on hypoxic pulmonary vasoconstriction of a lower [Hb] (71).

The causative nature of the relationship between the iron deficiency itself and exaggerated hypoxic pulmonary vasoconstriction is further attested by the significantly greater attenuation, by i.v. iron, of hypoxic pulmonary hypertension in the ID group. Again, it should be considered whether the lower [Hb] contributes, by increasing in some way the action of iron on the pulmonary vasculature. Radioactive isotope studies, however, indicate that when infused into profoundly ID individuals, iron is directed rapidly toward erythropoiesis (72). Thus the expected effect is to constrain iron availability for the pulmonary vasculature in the ID group, not increase it.

There is good reason to believe that the effects of iron deficiency on PASP are mediated by HIF. A collection of animal (7–10) and human (16, 18, 19) studies have confirmed the centrality of the HIF pathway in coordinating pulmonary vascular responses to hypoxia. In healthy humans, the time course of the rise in PASP seen during alveolar hypoxia is biphasic; an acute rise is seen rapidly and becomes maximal within minutes (48, 73) — a response too brisk to be mediated by a transcription factor pathway such as HIF. Subsequently, a second phase begins after approximately 40 minutes, continuing for several hours before plateauing (48, 74). During the second phase, PASP does not immediately fall back to baseline with euoxia, and a subsequent hypoxic stimulus will cause more marked hypoxic pulmonary vasoconstriction (45, 74). These are the hallmarks of pulmonary vascular acclimatization to hypoxia (75).

That the second phase of hypoxic pulmonary vasoconstriction brings with it a transient change in the properties of the pulmonary circulation suggests that hypoxia-regulated gene expression underlies the effect (76). In support of this conclusion, prolonged hypoxia in humans leads to pulmonary vascular remodeling (77), but in rodents with heterozygous deficiency of either HIF-1α (9) or HIF-2α (10) this phenomenon is greatly attenuated. Additionally, air-breathing rats fed an iron-restricted diet rapidly develop pulmonary arterial hypertension and right ventricular hypertrophy (78), in association with increased lung expression of HIF-1α and HIF-2α. Finally, the effect of i.v. iron to attenuate PASP elevation during hypoxia was evident in the present study during the second phase of hypoxic pulmonary vasoconstriction but not the first (Figure 2C); this is consistent with iron acting on hypoxia-regulated gene expression but not on those processes underlying acute hypoxic pulmonary vasoconstriction.

The hypoxic exposure on the first day induced a degree of acclimatization (Figure 2A). Experiments using similar sustained hypoxic exposures indicate that after return to euoxia for 3 hours, it is possible to demonstrate some residual PASP elevation and augmented hypoxic pulmonary vasoconstriction in response to a further acute hypoxic challenge (74). If a week of euoxia is allowed to pass, however, no elevation in euoxic PASP remains, and the magnitude of hypoxic pulmonary vasoconstriction returns completely to normal (45, 79). Thus, in the present study, an interval of a week or more was imposed between experimental days to ensure the hypoxic exposure on the first day would not affect findings during the second. Acute i.v. iron loading does not alter the first phase of hypoxic pulmonary vasoconstriction (62), so within each group, the near-identical euoxic PASPs and very similar acute PASP responses to hypoxia, on the second experimental day compared with the first, provide further evidence that 1 week was sufficient for any acclimatization to resolve.

Following i.v. iron, both groups exhibited a peak in PASP after 2 hours of hypoxia, after which PASP declined. In a previous study using a similar duration of hypoxia, this secondary decline was not observed (62). Instead, PASP was stable from 1 hour onward. In that study, the i.v. iron was administered as 200 mg iron sucrose over 105 minutes prior to hypoxia. This contrasts with the present study, in which up to 1000 mg ferric carboxymaltose was administered over 15 minutes before the exposure. Iron sucrose is cleared from the circulation into the tissues more rapidly than ferric carboxymaltose (72, 80). Thus the likely explanation for the difference between our findings and those of the previous study is that the ferric carboxymaltose, even at the higher dose, had less time to downregulate the mechanisms underlying the second phase of hypoxic pulmonary vasoconstriction, before the hypoxia was introduced. Thus in our study the second phase of hypoxic pulmonary vasoconstriction is evident in both groups on the second experimental day, before the effect of i.v. iron supervenes.

The technique used to determine PASP relies on the presence of a systolic tricuspid regurgitant jet. Advances in echocardiography reveal that most healthy individuals have physiological tricuspid regurgitation (TR), which is not considered a reflection of underlying pathology (81–83). Invasive measurements of pulmonary artery pressure correlate very well in a wide range of clinical settings with those obtained using echocardiography (84–88), and this holds true for healthy individuals rendered hypoxic (89). It is of interest whether healthy individuals without demonstrable TR are biologically different in some way. The prevalence of detectable TR has risen markedly with technological advances in echocardiography, without any accompanying change in the mean measured PASP (83, 90). This implies that the absence of TR does not simply reflect lower pressures in the pulmonary circulation. Furthermore, mortality is no different in individuals with and without TR sufficient to determine PASP (91), so if there are biological differences, their clinical significance is questionable. Associations between echocardiographic TR and characteristics of the left heart have been reported, including left atrial size, left ventricular end-diastolic diameter, and ejection fraction, though the size of the differences is small, and the direction of reported effects is not consistent (83, 90). Taking all this together, we cannot completely discount the possibly that pulmonary vascular behavior during hypoxia might differ, in some small way, in the minority of healthy individuals who do not have TR sufficient to measure PASP echocardiographically.

In addition to the pulmonary vasculature, we investigated the serum erythropoietin response to hypoxia. Interstitial fibroblasts in the renal cortex are responsible for secreting erythropoietin to regulate red cell production (92) but cannot detect [Hb] directly, and instead rely on the HIF pathway to sense local oxygen tension (93). Increased renal perfusion results in increased renal oxygen consumption due to the work of tubular reabsorption; thus renal tissue oxygen tension is largely independent of renal perfusion, and instead is mainly determined by blood oxygen content (94). This in turn is primarily a reflection of [Hb]. In this way, the kidney uses an oxygen-sensing pathway to sense [Hb]. As a consequence, a fall in blood oxygen content due to hypoxemia without any change in [Hb], as occurs acutely at high altitude (95), acts to stimulate erythropoietin production.

In the present study, both euoxic serum erythropoietin and the absolute rise in levels seen during sustained hypoxia were greater in the ID group, though the relative rises were not dissimilar. The marginally lower mean [Hb] in the ID group, coupled with multiple collinearity of serum ferritin, [Hb], and erythropoietin, precludes definite conclusions about causation. The differences observed are, though, certainly compatible with a direct effect of iron deficiency, and fit well with the observation that erythropoietin levels are considerably higher for a given [Hb] in the setting of iron deficiency anemia than anemias of other etiologies (96, 97). A direct action on erythropoietin secretion would also provide an explanation for observations from animal studies that, while polycythemia induced by transfusion attenuates hypoxic erythropoietin production, polycythemia induced by previous exposure to hypoxia — which will tend to cause iron deficiency because of the iron demand for erythropoiesis (95) — has the opposite effect (98).

In humans, HIF-2α is the predominant paralog controlling erythropoietin expression (93). The regulation of HIF-2α mRNA activity differs from that of HIF-1α mRNA; the former contains an iron-responsive element (IRE) whereas the latter does not. This IRE represses the translation of HIF-2α under conditions where iron is scarce; its importance is illustrated by mice deficient in iron regulatory protein 1, which develop polycythemia that is paradoxically stimulated by iron deficiency (8). These animals also exhibit spontaneous pulmonary hypertension. From the perspective of serum erythropoietin responses to hypoxia, there exists a tension between the effects of iron deficiency on HIF hydroxylase activity and those on HIF-2α mRNA translation, which may go some way to explaining the absence of a marked effect of acute iron loading on erythropoietin behavior in the present study.

Hepcidin is recognized as the major hormone regulating iron homeostasis. It acts to lower serum iron levels by promoting the degradation of ferroportin, the only mammalian cellular iron export protein so far identified (99). A rise in serum iron is signaled via a mechanism involving transferrin receptors on the surface of hepatocytes (100). Hepcidin secreted in response impairs the ability of cells of the reticuloendothelial system and duodenal enterocytes to export iron. Hepcidin is not only regulated by serum iron levels; erythropoietic drive and the innate immune system are other important factors (101). The gene encoding hepcidin is transcriptionally regulated by IL-6; thus inflammatory stimuli lead to hypoferremia (102) and reduced iron availability for pathogens. Unfortunately, this contributes to iron sequestration in chronic inflammatory conditions and the anemia of chronic disease (40). The complex interplay between iron homeostasis, hypoxia, and inflammation makes it challenging to establish causation, so we sought to study profoundly iron-deficient but otherwise healthy individuals to provide mechanistic clarity. The iron deficiency seen in our participants was naturally occurring absolute iron deficiency — due to factors such as blood donation, diet, and menstrual blood loss — confirmed by the profoundly suppressed hepcidin level in the ID group.

Hepcidin expression is suppressed indirectly by hypoxia via stimulation of erythropoiesis (103). This mechanism does not depend on a fall in serum iron from increased erythrocyte uptake (104) but depends instead on a putative factor named erythroferrone produced by the bone marrow (105). Interestingly, we found that hepcidin rose in both groups on both study days during each 6-hour hypoxic exposure. Over longer periods hypoxia clearly suppresses hepcidin (95), but an early transient rise in hepcidin during hypoxia was also suggested in a previous study (104). Given that IL-6 rose slightly during hypoxia, to a similar extent in both groups on both days, it may be that acute hypoxia generates an inflammatory signal that drives hepcidin expression over hours, before the suppressive effect of erythropoietic drive and erythroferrone production supervenes. If so, this has implications for diseases characterized by intermittent as opposed to chronic hypoxia. Interestingly, iron sequestration driven by elevated hepcidin has recently been reported in obstructive sleep apnea (106, 107), one example of such a condition.

The magnitude of the rise in hepcidin was much more substantial in the IR group, presumably because of a potent suppressive effect of low serum iron on hepcidin secretion in the ID participants. When i.v. iron was given, hepcidin levels rose more markedly, as expected, in response to hyperferremia. The rise was again constrained in the ID group, suggesting that existing tissue iron depletion still acts as a strong negative regulatory signal even when serum iron levels are acutely elevated. With this in mind, it is interesting to note that in Ethiopian highlanders with elevated [Hb], hepcidin is not heavily suppressed despite exposure to chronic steady-state hypoxia; iron demand and body iron stores instead appear to be the primary regulators of circulating hepcidin in this setting (108). Equally, venesection of Peruvian high-altitude residents suffering from chronic mountain sickness — a condition in which polycythemia, hypoxemia, and pulmonary hypertension are features (109) — brings about a very rapid fall in circulating hepcidin levels, consistent with an erythroid regulator signaling the tension between erythropoietic drive and iron supply (110).

Studies of high-altitude populations also demonstrate that polycythemia is not an inevitable consequence of chronic exposure to hypobaric hypoxia. For example, Tibetans resident at 4000 m show a similar [Hb] to those of US sea-level residents (111). This group is also remarkable for exhibiting relatively normal pulmonary artery pressures at altitude, and very modest hypoxic pulmonary vasoconstriction in response to a further fall in ambient oxygen tension (112), despite showing very marked ventilatory responses to hypoxia (113). As already discussed, it may be that some of this protection against high-altitude hypoxic pulmonary hypertension is explained by the lower [Hb] itself, and conversely that much of the pulmonary hypertension seen in individuals in whom chronic mountain sickness develops is driven by polycythemia (109). Putting aside these complexities, it is notable that different high-altitude populations display very different combinations of traits, which include metabolic, cerebrovascular, and reproductive characteristics in addition to the pulmonary vascular, ventilatory, and hematological features already discussed (114). Evidence increasingly implicates differences in genes encoding proteins involved in oxygen and iron homeostasis, particularly those of the HIF pathway (19–21, 115–117).

In the present study, no differences were seen in ventilation between the groups, nor was a discernible acute effect of i.v. iron supplementation on ventilation evident. These findings mirror those of a previous study in which acute iron chelation did not affect ventilation (61). As with the kidney, the apparent insensitivity of the carotid body to chronic differences in iron bioavailability and acute iron loading may be explained by different relative contributions from the multiple HIF paralogs (2, 22) or perhaps differences in iron transport mechanisms compared with the pulmonary vasculature.

In conclusion, our study provides the first evidence, to our knowledge, of a clinically meaningful effect of iron deficiency on pulmonary vascular biology. It implies that iron status modulates the HIF pathway in vivo in a significant way, and it confirms the potential of manipulation of iron homeostasis as a tool to treat diseases in which hypoxia plays a role. This is particularly the case for conditions in which pulmonary arterial hypertension is a feature, including both congestive cardiac failure (118) and chronic lung disease (119), but is by no means limited to such conditions; hypoxia-sensitive pathways are also central to angiogenesis, neoplasia, and human reproduction. Given the extensive operation of HIF hydroxylase pathways in human biology, and the abundance and pleiotropic actions of other iron- and 2-oxoglutatrate–dependent dioxygenases, our findings raise the possibility that alterations in oxygen sensing and signaling could underlie deleterious effects of iron deficiency in a wide range of situations.

## Methods

This was a prospective, nonrandomized controlled clinical study with participants blinded to intervention order. We recruited otherwise healthy adults with absolute iron deficiency. Iron-replete age-matched (to within a decade) and sex-matched volunteers served as controls. Participants were studied on 2 occasions, a week or more apart, during a sustained hypoxic exposure.

### Eligibility criteria.

Inclusion criteria were: ability to give informed consent; aged at least 18 years; and presence of detectable TR on transthoracic Doppler echocardiography enabling measurement of PASP. For recruitment to the ID group, both a serum ferritin ≤ 15 μg/l and a transferrin saturation less than 16% were required. For IR volunteers, these values were ≥ 20 μg/l and ≥ 20%, respectively. These values were not intended to reflect a universally accepted definition of iron deficiency, since there is none (39); their primary function was to generate 2 groups differing significantly in iron status. Exclusion criteria were: hemoglobin less than 80 g/l; hemoglobinopathy; serum ferritin greater than 300 μg/l; SpO_2_ less than 94%; iron supplementation or blood transfusion within 6 weeks; pregnancy or breastfeeding; and any significant comorbidity potentially affecting hematinics, pulmonary vascular responses to hypoxia, or ventilation (including inflammatory conditions and those causing intermittent hypoxia, such as obstructive sleep apnea). Volunteers were also excluded if recently exposed to altitude greater than 2,500 m or air travel longer than 4 hours.

### Participant recruitment and matching.

During the period of recruitment between February 2013 and April 2014, blood donors in Oxfordshire, UK, were offered information about the study if below the hemoglobin threshold to donate, since such individuals frequently have iron deficiency (120). Advertisements were placed concurrently for controls. Volunteers attended a screening visit conducted by a physician including medical history, examination, spirometry (MicroLab, CareFusion, UK), transthoracic echocardiography (Vivid-q, GE Healthcare), venous blood sampling, and a brief hypoxic exposure, to establish eligibility and familiarize participants with the study procedures. Before data analysis, ID and IR participants were matched in pairs according to sex and age, since both may affect pulmonary vascular physiology (83, 121–123).

### Exposure to hypoxia.

Each study day entailed a 6-hour eucapnic hypoxic exposure (oxygen end-tidal partial pressure 55 mmHg) in a normobaric chamber. The apparatus (124) included a computerized system for continuously monitoring end-tidal gases via a nasal cannula. Continuous electrocardiography and pulse oximetry were performed, and ventilation monitored by computerized analysis of gas entrained from the nasal cannula. Nitrogen, CO_2_, and oxygen were introduced via a rapid fan-mixing system, and CO_2_ removed by passing of ambient gas through a soda-lime filtration system, permitting inspired gas concentrations to be controlled tightly. Participants were provided with light refreshment ad libitum, and were able to move around, enjoy audiovisual entertainment, and leave the chamber briefly to use the lavatory if required.

### Blood sampling and infusions.

On the first study day, immediately before commencement of the hypoxic exposure, 0.9% saline was administered i.v., and on the second, 15 mg/kg (maximum 1 g) ferric carboxymaltose (Ferinject, Vifor Pharma) was added to an appropriate volume of 0.9% saline; each infusion was of 250 ml total volume and given over 15 minutes at a rate of 16.7 ml/min. Infusion of 0.9% saline in healthy individuals at rates and volumes very considerably in excess of this does not produce significant effects on echocardiographic measurements of pulmonary or systemic circulatory hemodynamics (125). Though it was not possible to randomize the order of infusions, participants were blindfolded during administration and not told that the infusions would follow a consistent order. Venous blood was sampled before each infusion and at 6 hours. Routine assays were performed by a university hospital laboratory. Serum and plasma were obtained by centrifugation and frozen at –80°C. Erythropoietin, sTfR, IL-6 (all Quantikine, R&D Systems), and hepcidin (Hepcidin-25 EIA kit, Bachem, Peninsula Laboratories) were measured in triplicate by ELISA in accordance with the manufacturer’s instructions.

### Doppler echocardiography.

Throughout each hypoxic exposure, PASP and CO were measured echocardiographically (81–89). Participants rested comfortably on a customized couch in the left lateral position facing the operator while the maximum systolic pressure across the tricuspid valve (ΔPmax) was determined from an apical 4-chamber view of the heart using continuous wave Doppler. Stroke volume (SV) was measured from the velocity-time integral of left ventricular outflow tract (LVOT) blood flow using pulsed-wave Doppler in an apical 5-chamber view, the LVOT diameter having been determined from a parasternal long-axis view. CO was determined by multiplication of SV and heart rate. PASP was calculated by addition of 5 mmHg, as an estimate of right atrial pressure, to ΔPmax (44, 45, 62, 75).

### Statistics.

The prespecified primary outcome measure was rise in PASP over the initial 6-hour hypoxic exposure in ID compared with IR participants. The study was designed to have 80% power to detect a difference in the rise in PASP between groups of 4 mmHg with a 2-sided significance level of 0.05. Data were analyzed using SPSS (version 20, IBM). Mixed-effects modeling was used to determine effects of hypoxia and iron infusion between and within groups. Group characteristics were compared using Student’s *t* test, or the Mann-Whitney *U* test for non-normally distributed data. Spearman’s rank correlation coefficient was calculated for assessment of correlation between variables within groups. In all cases, *P* less than 0.05 was taken to be statistically significant.

### Study approval.

The study was conducted in accordance with the Declaration of Helsinki and received ethical approval from the National Research Ethics Service National Health Service (NHS) South Central Portsmouth Research Ethics Committee (reference: 12/SC/0710). The study sponsor was the University of Oxford. All participants provided written informed consent.

## Author contributions

MCF, AHN, PJR, and PAR conceived and designed the study. MCF, HYC, AHN, DJR, and KLD recruited participants. MCF, HYC, KAP, and MKC acquired the data. MCF, KAP, MKC, DJR, KLD, and PAR analyzed and interpreted the data. MCF drafted the manuscript. KLD, DJR, PJR, and PAR revised the manuscript critically for important intellectual content. All authors approved the final version of the manuscript.

## Supplementary Material

ICMJE disclosure forms

## Figures and Tables

**Table 2:**
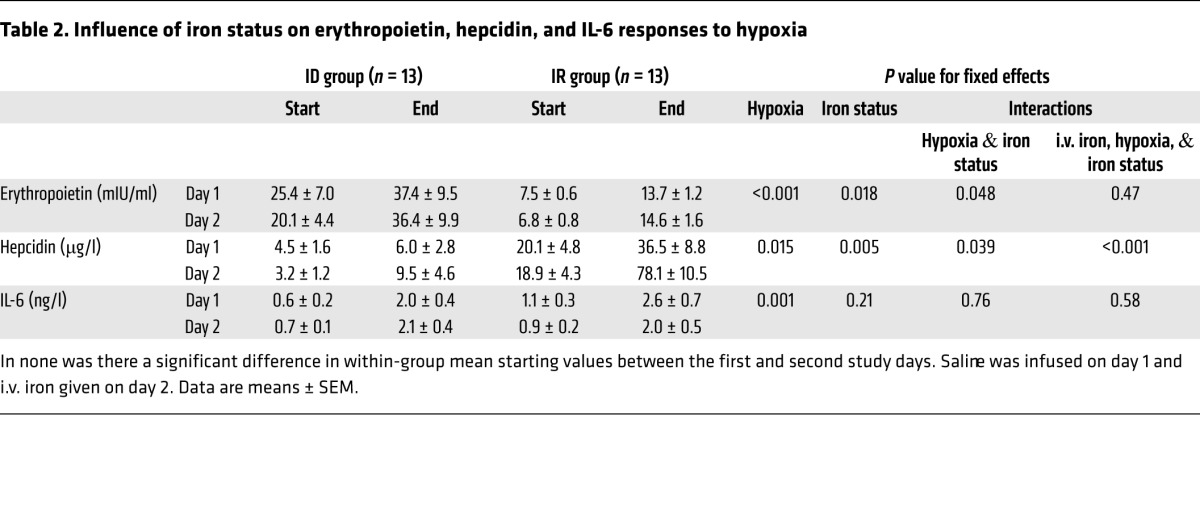
Influence of iron status on erythropoietin, hepcidin, and IL-6 responses to hypoxia

**Table 1:**
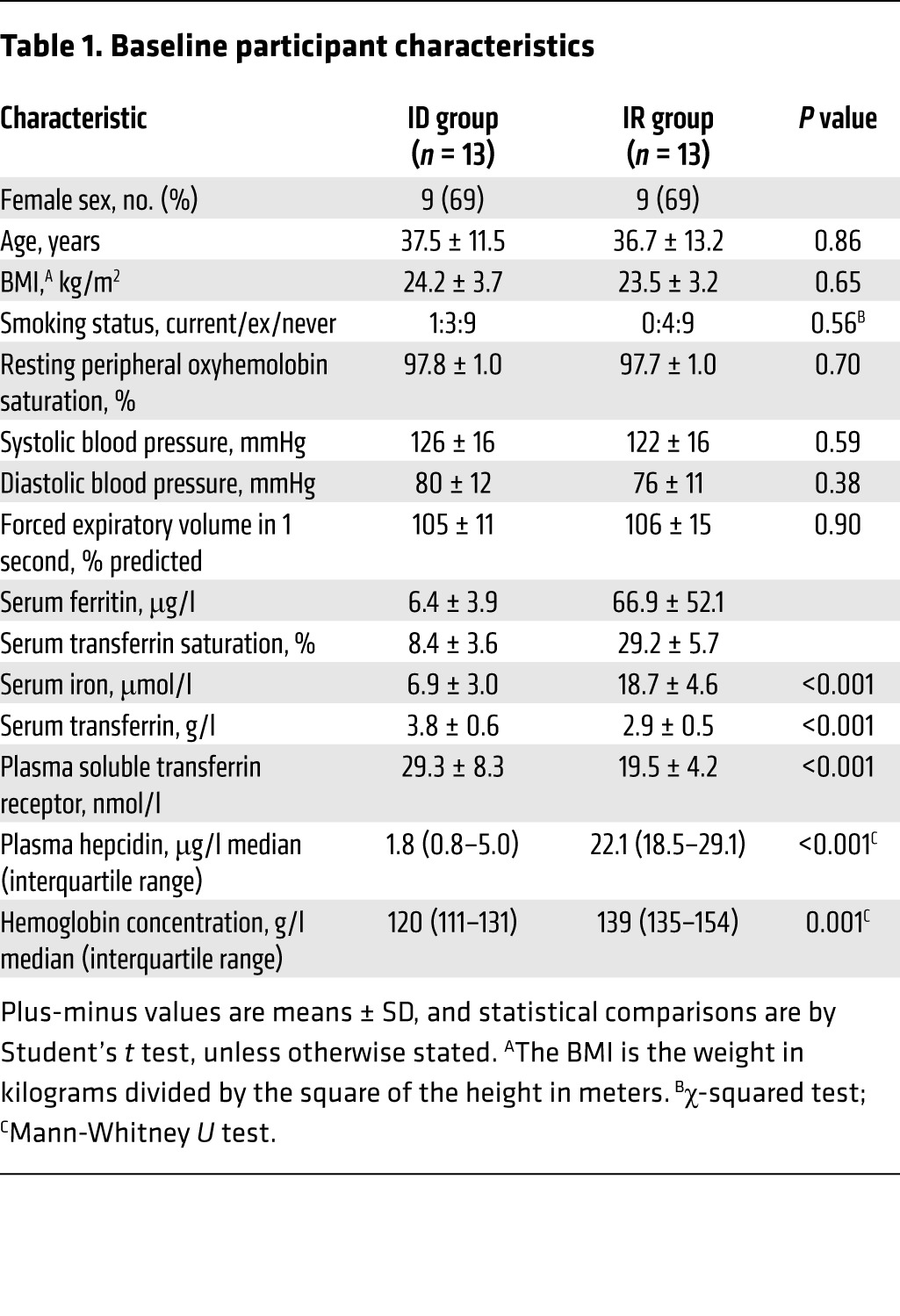
Baseline participant characteristics
